# Interleukin-6 drives endothelial glycocalyx damage in COVID-19 and bacterial sepsis

**DOI:** 10.1007/s10456-024-09916-w

**Published:** 2024-04-10

**Authors:** Carolin Christina Drost, Alexandros Rovas, Irina Osiaevi, Klaus Schughart, Alexander Lukasz, Wolfgang A. Linke, Hermann Pavenstädt, Philipp Kümpers

**Affiliations:** 1 Department of Medicine D, Division of General Internal and Emergency Medicine, Nephrology, and Rheumatology, University Hospital Münster, Albert-Schweitzer-Campus 1, 48149 Münster, Germany; 2 Department of Medicine A, Hematology, Oncology and Pulmonary Medicine, University Hospital Muenster, 48149 Muenster, Germany; 3 Department of Microbiology, Immunology and Biochemistry, University of Tennessee Health Science Center, Memphis, TN USA; 4 Institute of Virology Münster, University of Münster, Münster, Germany; 5 Institute of Physiology II, University Hospital Münster, Robert-Koch-Straße 27b, 48149 Münster, Germany

**Keywords:** Sepsis, COVID-19, Heparanase, Sublingual microscopy, Endothelial glycocalyx

## Abstract

Damage of the endothelial glycocalyx (eGC) plays a central role in the development of vascular hyperpermeability and organ damage during systemic inflammation. However, the specific signalling pathways for eGC damage remain poorly defined. Aim of this study was to combine sublingual video-microscopy, plasma proteomics and live cell imaging to uncover further pathways of eGC damage in patients with coronavirus disease 2019 (COVID-19) or bacterial sepsis. This secondary analysis of the prospective multicenter MICROCODE study included 22 patients with COVID-19 and 43 patients with bacterial sepsis admitted to intermediate or intensive care units and 10 healthy controls. Interleukin-6 (IL-6) was strongly associated with damaged eGC and correlated both with eGC dimensions (r_s_=0.36, *p* = 0.0015) and circulating eGC biomarkers. In vitro, IL-6 reduced eGC height and coverage, which was inhibited by blocking IL-6 signalling with the anti-IL-6 receptor antibody tocilizumab or the Janus kinase inhibitor tofacitinib. Exposure of endothelial cells to 5% serum from COVID-19 or sepsis patients resulted in a significant decrease in eGC height, which was attenuated by co-incubation with tocilizumab. In an external COVID-19 cohort of 219 patients from Massachusetts General Hospital, a previously identified proteomic eGC signature correlated with IL-6 (r_s_=-0.58, *p* < 0.0001) and predicted the combined endpoint of 28-day mortality and/or intubation (ROC-AUC: 0.86 [95% CI: 0.81–0.91], *p* < 0.001). The data suggest that IL-6 may significantly drive eGC damage in COVID-19 and bacterial sepsis. Our findings provide valuable insights into pathomechanisms of vascular dysfunction during systemic inflammation and highlight the need for further in vivo studies.

**Supplementary Information:**

The online version contains supplementary material available at 10.1007/s10456-024-09916-w.

## Introduction

The vascular endothelium is lined by a gel-like matrix of highly sulphated glycosaminoglycans (sGAGs) attached to core proteoglycans, the so-called endothelial glycocalyx (eGC). This fragile structure shields the endothelium from pathogenic insults and plays a key role in maintaining microcirculatory homeostasis. Specifically, the eGC acts as a negatively charged “firewall” to reduce leukocyte-endothelial interactions [1–4]. Its carbohydrate-rich matrix provides resistance to water permeability and contributes to the proportion of albumin molecules “reflected” back into plasma by the vessel wall [5, 6]. In addition, the glycocalyx contributes to the regulation of the redox state and is critically involved in mediating shear-induced nitric oxide release and physiologic anticoagulation [2, 7, 8]. Inflammation-induced eGC dysfunction leads to vascular hyperpermeability resulting in oedema formation and organ damage in critically ill and particularly septic patients [9, 10].

The ultimate shared pathway of eGC damage, particularly in bacterial sepsis and COVID-19, seems to involve the activation and release of heparanase (HPSE), a heparan sulphate (HS)-degrading enzyme that is unique to mammals and breaks down HS chains from HS proteoglycans found in the glycocalyx [9–11]. However, upstream of HPSE upregulation and release, only a few specific signalling pathways have been identified, all of which are pathophysiologically relevant in sepsis. These include the angiopoietin-1/Tie2 ligand receptor system, tumor necrosis factor (TNF)-alpha/TNF receptor signalling and toll-like receptor (TLR)-2 and − 4 signalling [2, 3, 12–14].

In this study, we employed sublingual video-microscopy imaging and plasma proteomics to uncover further pathways of eGC damage. The most promising mediator candidate – Interleukin-6 (IL-6) – was subsequently tested for causality by atomic force microscopy in an established endothelial cell (EC) culture system.

## Materials and methods

### Study design and study population

The clinical part of this study is a secondary analysis of a previous multicenter, prospective, observational, cross-sectional study conducted from May to June 2020 at the University Hospital Münster and three local academic teaching hospitals [15]. The main finding of this study was that the microvascular and proteome signatures of both COVID-19 and bacterial sepsis were very similar and showed almost the same changes compared to healthy controls. We therefore pooled COVID-19 and bacterial sepsis patients in some exploratory analyses in the current study.

After written informed consent was obtained, adult hospitalized patients requiring intensive care (ICU-critical disease) or intermediate care (IMC-moderate/severe disease) because of COVID-19 infection or confirmed bacterial sepsis (sepsis-3 definition) [16] were prospectively enrolled in a non-consecutive manner. Sublingual video-microscopy was performed at the same time as blood sampling. Plasma samples were collected, centrifuged, and stored at -80 °C until analysis. Exclusion criteria were pregnancy or local inflammation of the oral mucosa. None of the patients received therapy specifically targeting IL-6. Ten apparently healthy, randomly selected age-matched volunteers served as controls. Three randomly selected serum samples with high IL-6 levels (from the third IL-6 tertile) of each COVID-19 and sepsis were used for in vitro experiments. This study was approved by the local ethics committee (amendments of 2016–073-f-S) and was conducted in accordance with the Declaration of Helsinki.

### In vivo assessment of sublingual microcirculation and glycocalyx dimensions

A sidestream dark field camera (CapiScope HVCS, KK Technology, Honiton, UK) coupled with GlycoCheck™ software (Microvascular Health Solutions Inc., Alpine, UT, USA) was used to visualize passing red blood cell (RBC) flow in the sublingual microvasculature (microvessel diameter 4–25 μm) at the bedside as previously described in detail [17, 18]. Based on the RBC dynamics in the valid vessel segments, the software calculates the following variables, which have been successfully validated in the past [12, 17–19]:

*Perfused Boundary Region* (PBR, in µm) expresses the dynamic lateral movement of RBCs into the permeable part of the endothelial glycocalyx layer, an inverse parameter of the endothelial glycocalyx thickness. The higher the PBR values, the thinner the glycocalyx (Supplemental Fig. 1).

*Capillary density* (in 10^− 2^mm/mm^2^) was defined as the vascular density of vessels with a diameter ≤ the diameter of a single erythrocyte (diameter ~ 7–8 μm [20]; capillary density diameter 4–7 μm).

*RBC velocity* (in µm/sec) can be automatically determined in individual vessel segments by cross-correlating RBC longitudinal intensity profiles between frames of recorded videos.

### Targeted plasma proteomics and circulating glycocalyx markers

The “Inflammation1” and “Cardiovascular2” proteomic panels from Olink (Sweden) each contained 92 proteins. Seven proteins (including IL-6) were common to both panels. A total of 184 proteins in 76 samples (COVID-19, bacterial sepsis, healthy) were measured in one batch to avoid technical variation. In brief, two specific oligonucleotide-labelled antibodies per protein (‘probes’) were used in the Olink proximity extension assay. When the two probes were in close proximity to each other, a new PCR target sequence was formed via a proximity dependent DNA polymerisation event. The resulting sequence was then detected and quantified by standard real-time quantitative PCR as previously reported [21]. Measurements were performed in triplicates. Results were expressed in arbitrary units. The proteins included in each panel, measurement details and validation data are available online (www.olink.com/downloads).

Plasma levels of the glycocalyx core protein syndecan-1 (Diaclone, Besançon, France) and hyaluronic acid (Echelon Biosciences Inc., Salt Lake City, UT, USA) were measured using commercially available enzyme-linked immunosorbent assay kits according to the manufacturer’s instructions.

### External validation set ─ Massachusetts General Hospital COVID-19 cohort

Some findings were validated in a public database of adult COVID-19 patients admitted to the Massachusetts General Hospital (MGH, Boston, Massachusetts, USA, https://www.olink.com/mgh-covid-study), which annotates proteomic and outcome data [22]. Inclusion criteria were clinical concern for COVID-19 on admission to the emergency department and acute respiratory distress with at least one of the following: respiratory rate ≥ 22 breaths/minute; oxygen saturation ≤ 92% on room air; need for supplemental oxygen; positive pressure ventilation. The primary endpoint of the study was a composite endpoint of 28-day mortality and/or intubation, and a total of 219 blood samples collected on day 3 were analyzed in relation to the primary endpoint.

### Cell culture and reagents

Cells of the human umbilical vein endothelial cell (HUVEC) line EA.hy926 were grown in DMEM (Gibco™; Cat# 52100047) supplemented with 10% fetal calf serum (FCS, SigmaAldrich) and 1% penicillin/streptomycin (Biochrom; Cat# A2212) at 37 °C in a 5% CO_2_ enriched environment for a minimum of 3 days until reaching confluence.

Experiments were conducted in (4-(2-hydroxyethyl)-1-piperazineethanesulfonic acid) (HEPES) buffer (140 mM NaCl, 5 mM KCl, 1 mM CaCl_2_, 1 mM MgCl_2_, 5 mM glucose, 10 mM HEPES) supplemented with 1% FCS and incubation times of 60 min if not otherwise stated. IL-6 (#200-06) and sIL-6R (#200-06RC) were purchased from PeproTech (Thermo Fisher Scientific, Germany), Heparin (#H3149) and Tofacitinibcitrate (#PZ0017) from Sigma-Aldrich (Germany), and Tocilizumab (EU: RoActemra®, US: Actemra®) from Roche (Germany).

### 1,9-Dimethylmethylene blue assay

To measure the amount of sGAGs in the supernatant samples, the 1,9-Dimethylmethylene Blue (DMMB) assay was performed as previously described [23, 24]. Briefly, after incubation, the cell supernatant was collected and concentrated by centrifugation in Microcon-10 kDa centrifugal filter units (Merck; Cat# MRCPRT010) for 20 min at 14,000 x g. 50 µl of the concentrate were transferred in duplicates to a 96-well plate (Greiner bio-one; Cat# 655,101). 200 µl of the DMMB buffer was added per well and the absorbance was immediately read at 525 and 590 nm (Tecan, Infinite M200). A standard curve with chondroitin sulfate-4 served as a control. Data were presented standardized to the corresponding control condition.

### Atomic force microscopy

Quantification of eGC thickness in vitro was performed by atomic force microscopy (AFM) nanoindentation technique using a Nanoscope V multimode AFM (Veeco, Mannheim, Germany) as previously described in detail [3, 12]. The measurement was performed in a liquid chamber at 37 °C in HEPES buffer supplemented with 1% FCS. Periodic indentation and deflection of the triangular cantilever with a mounted 10 μm spherical tip (Novascan Technologies, Boone, North Carolina, United States) - spring constant 10 pN/nm – was detected by a laser beam. The eGC thickness of the indented area was calculated from the resulting force versus distance curve. By measuring at least 24 cells per group/condition, AFM can detect significant differences of at least 15% between three groups with a power of 95% power (G*Power 3.1). Data were presented standardized to the corresponding control condition.

### Immunofluorescence

Heparan sulphate (HS) staining was performed essentially as described in previous studies [11, 14, 25]. Cells were fixed with 2% paraformaldehyde plus 0.1% glutaraldehyde followed by overnight incubation with primary antibody (Amsbio, Ab heparan sulfate, Cat# 370255-1) and secondary antibody (Jackson ImmunoResearch Labs, Alexa Fluor 488 goat anti-mouse IgG antibody, Cat# 115-545-146) and 4,6-diamidino-2-phenylindole dihydrochloride (DAPI). Images were captured with a Leica DMI 6000B-CS/TCS SP8 laser confocal microscope (objective: HC PL APO CS2 63 × /1.40 oil; Leica, Wetzlar, Germany) and analyzed using LasX software (Leica, Wetzlar, Germany) and ImageJ software (version 1.51p 22, National Institutes of Health, United States).

### Statistics

Data were presented as indicated with median ± interquartile range (IQR) or mean ± standard error of mean (SEM), unless otherwise noted. Differences between two groups were calculated by Mann-Whitney U test or chi-square, as appropriate. Comparisons between ≥ 3 groups were performed with Kruskal-Wallis test with Dunn’s post-hoc test. The Spearman correlation coefficient (r_s_) was used to assess correlations between variables. For the AFM and DMMB experiments, nested ANOVA with Tukey’s post-hoc test was used to account for the number of observations per experiment and the number of experiments.

Analysis and visualization of protein expression data was performed using the R software package (version 4.2.1) [26]. After quality control, one Covid-19 ICU outlier sample was excluded (very low median expression level compared to all other samples). For identification of differentially expressed proteins (DEPs), the Limma package (version 3.52.4 [27, 28]), was used with the design: model.matrix(~ 0 + group). For the contrast of infected versus healthy controls, groups were: all infected (bacterial sepsis and COVID-19 combined, *n* = 65) and healthy controls. For the other comparisons, groups were: healthy controls, COVID-19 ICU, bacterial sepsis ICU. DEPs were identified based on an adjusted p-value of < 0.05 and exhibiting more than a 1.5-fold (log_2_ = 0.5849625) difference in expression levels. Multiple testing adjusted p-value were calculated according to Benjamini and Hochberg [29]. Volcano plots were generated with the package EnhancedVolcano, version 1.14.0 [30].

All tests were two-tailed and significance was accepted at *p* < 0.05. GraphPad Prism version 9 (GraphPad Prism Software Inc, San Diego, California, USA) and SPSS 29 (IBM, Armonk, New York, NY, USA) were used for further data analysis and figure preparation.

## Results

Our cohort consisted of 43 patients with bacterial sepsis and 22 with COVID-19. There were no significant differences between median [IQR] age (68 [57–79] vs. 63 [53–76] years, *p* = 0.12), sex (*p* = 0.14), or disease severity (Sequential Organ Failure Assessment (SOFA) score 9 [4-12] vs. 6 [2-12], *p* = 0.22) in the two groups (Table 1). ICU patients had a higher SOFA score than IMC patients (10 [6-13] vs. 2 [0.5-3], *p* < 0.0001) and were ventilated in 66,7% of cases. IMC patients were more likely to be female (47.1% vs. 18.8%, *p* = 0.023), but did not differ from ICU patients in terms of age (64 [55-79.5] vs. 64.5 [56.3–76.5] years, *p* = 0.69) (Supplemental Table 1).

**Table 1:** Baseline characteristics, confer primary analysis in Rovas et al. [15]

Variables	Healthy Controls	Bacterial sepsis	COVID-19	p value*
Number of participants (n)	10	43	22	-
Female sex (n; (%))	7 (70)	14 (32.6)	3 (13.6)	0.14
Age (years, median (IQR))	51 (27–69)	68 (57–79)	63 (53–76)	0.12
BMI (kg/m^2^, median (IQR))	23 (21.5–25.8)	25.3 (21.1–27.7)	26.5 (23.4–30.1)	0.15
Charlson Comorbidity Index (points, median (IQR))	-	2 (1–3)	1 (0–3)	0.14
ICU (n; %)	-	33 (76.7)	15 (68.2)	0.55
SOFA score (points, median (IQR))	-	9 (4–12)	6 (2–12)	0.22
Mechanical ventilation (n; %)	-	19 (44.2)	13 (59.1)	0.30
Inhospital mortality (n; %)	-	13 (30.2)	6 (27.3)	> 0.99
MAP (mmHg)	92.3 (89.2–99.4)	73.7 (66.7–87.3)	78.2 (71.9–90.2)	0.29
**Sublingual video-microscopy** (median (IQR))				
PBR_4 − 25 μm_ (µm)	2.23 (2.1–2.34)	2.46 (2.33–2.62)	2.31 (2.15–2.51)	0.012
RBCV_4 − 7 μm_ (µm/sec)	100 (88–118)	92 (78–108)	90 (79–109)	0.75
Density_4 − 7 μm_ (10^− 2^mm/mm^2^)	118.9 (81.7–132.1)	54.1 (35.8–88.1)	56.2 (37.0–98.0)	0.91
**Laboratory data** (median (IQR))				
CRP (mg/dl)	0.5	21.6 (12.8–31.8)	12.2 (4.5–21.9)	0.02
PCT (ng/ml)	0.05	7.3 (0.7–46.7)	0.6 (0.1–3.2)	< 0.001
Creatinine (mg/dl)	0.85 (0.68–0.95)	1.9 (1.2–3.1)	0.8 (0.6–1.5)	0.003
IL-6 (ng/ml)	2	355 (85–1101)	62 (24–153)	0.0004

*p-value was calculated between bacterial sepsis and COVID-19 cohort. Analysis was performed with Mann-Whitney test or Chi-square test as appropriate. BMI = Body mass index, CRP = C-reactive protein, IQR = interquartile range, MAP = Mean arterial pressure, PCT = Procalcitonin, SOFA score = Sequential Organ Failure Assessment score, PBR = Perfused boundary region, RBCV = Red blood cell velocity, IL-6 = Interleukin-6

### Proteome analysis identifies IL-6 as potential mediator of eGC damage in inflammation

**Fig. 1:**
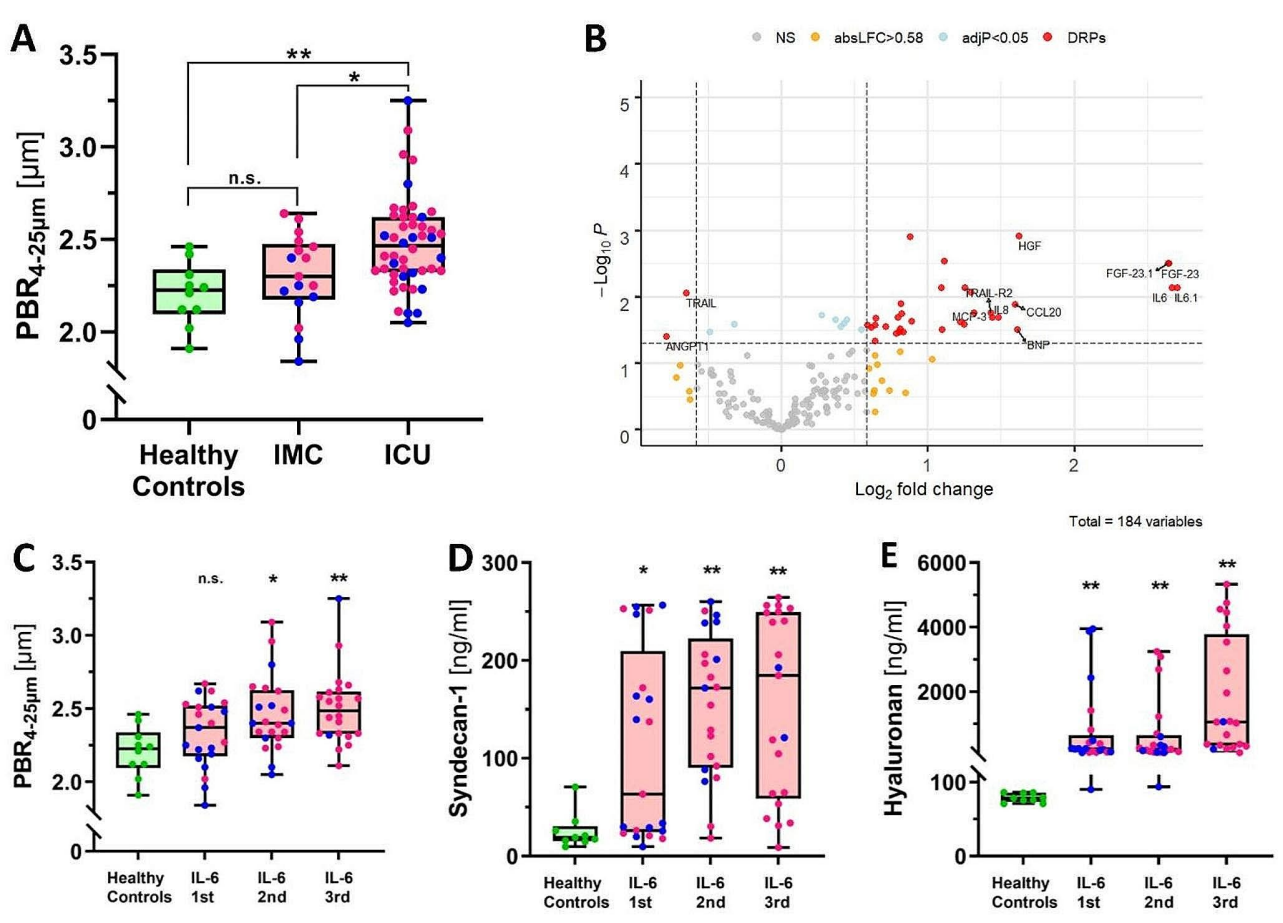
Proteome analysis identifies IL-6 as potential mediator of eGC damage in COVID-19 and bacterial sepsis. (**A**) Box plots (median ± IQR) of perfused boundary region (PBR) values from sublingual video-microscopy in healthy controls, intermediate care (IMC) and intensive care (ICU) patients. (**B**) Volcano plot showing log_2_-fold changes and adjusted p-values of differentially regulated proteins (DRPs) for low vs. high PBR groups (using a cut-off of 2.25 μm). As IL-6 is included in both proteomic panels, it appears twice. Box plots of (**C**) PBR, (**D**) circulating syndecan-1 (available in 72 subjects) and (**E**) circulating hyaluronan (available in 72 subjects) in healthy controls vs. patients (IL-6-derived tertiles, first tertile = lowest IL-6 levels). Individual values are shown as green dots = healthy controls; blue dots = COVID-19; pink dots = sepsis. Significance was tested with Mann-Whitney (also see Supplemental Table 1) or Kruskal-Wallis and Dunn’s post-hoc test against healthy controls. *=*p* < 0.05, **=*p* < 0.01

ICU patients with either sepsis or COVID-19 had significantly higher PBR_4 − 25 μm_ values (i.e., thinner endothelial glycocalyx) than healthy controls or IMC patients, respectively (Fig. 1A, Supplemental Table 1). A pooled analysis including COVID-19 and bacterial sepsis patients showed that PBR values correlated with the SOFA score (r_s_ = 0.28, *p* = 0.016), indicating that disease severity correlates with eGC damage.

To identify potential mediators of glycocalyx damage, we performed differentially expressed protein (DEP) analysis after dividing participants into those with intact vs. damaged eGC (Supplemental Fig. 2). When compared to participants with intact eGC, there were 31 up- and 2 downregulated DEPs in participants with damaged eGC. Of these, IL-6 showed by far the largest log_2_ fold change of all 177 proteins (Fig. 1B). This finding was consistently reproducible and even more pronounced when classification was based on disease entity rather than eGC, suggesting that IL-6 upregulation was relevant in both COVID-19 and sepsis (Supplemental Fig. 3).

Median IL-6 levels measured at routine laboratory were significantly higher in patients with damaged eGC than in those with intact eGC (131 [69–826] vs. 30 [2–90] ng/ml, *p* = 0.0003; Supplemental Table 2). Furthermore, IL-6 levels correlated with the PBR (r_s_=0.36, *p* = 0.0015) and circulating biomarkers of eGC damage (syndecan-1: r_s_ = 0.41, *p* = 0.0004; hyaluronan: r_s_ = 0.58, *p* < 0.0001). When visualized by overall IL-6 tertiles, PBR, syndecan-1 and hyaluronan showed a steady increase (Fig. 1C-E). Consistent with previous work, showing an uncoupling of PBR (eGC integrity) and microvascular perfusion [17, 19], IL-6 levels were only weakly associated with capillary density (r_s_ = -0.27, *p* = 0.018) (Supplemental Table 2, Supplemental Fig. 4).

### IL-6 signalling causes eGC damage in vitro

In classical IL-6 signalling, IL-6 binds to the membrane-bound IL-6 receptor α subunit (hereafter mIL-6R) and the glycoprotein 130 (gp130) signal-transducing subunit. In contrast, in IL-6 trans-signalling, complexes of IL-6 and the soluble form of the IL-6 receptor (sIL-6R) signal via (ubiquitously expressed) membrane-bound gp130. HUVECs express both receptors, gp 130 and small amounts of mIL-6R [31, 32].

**Fig. 2:**
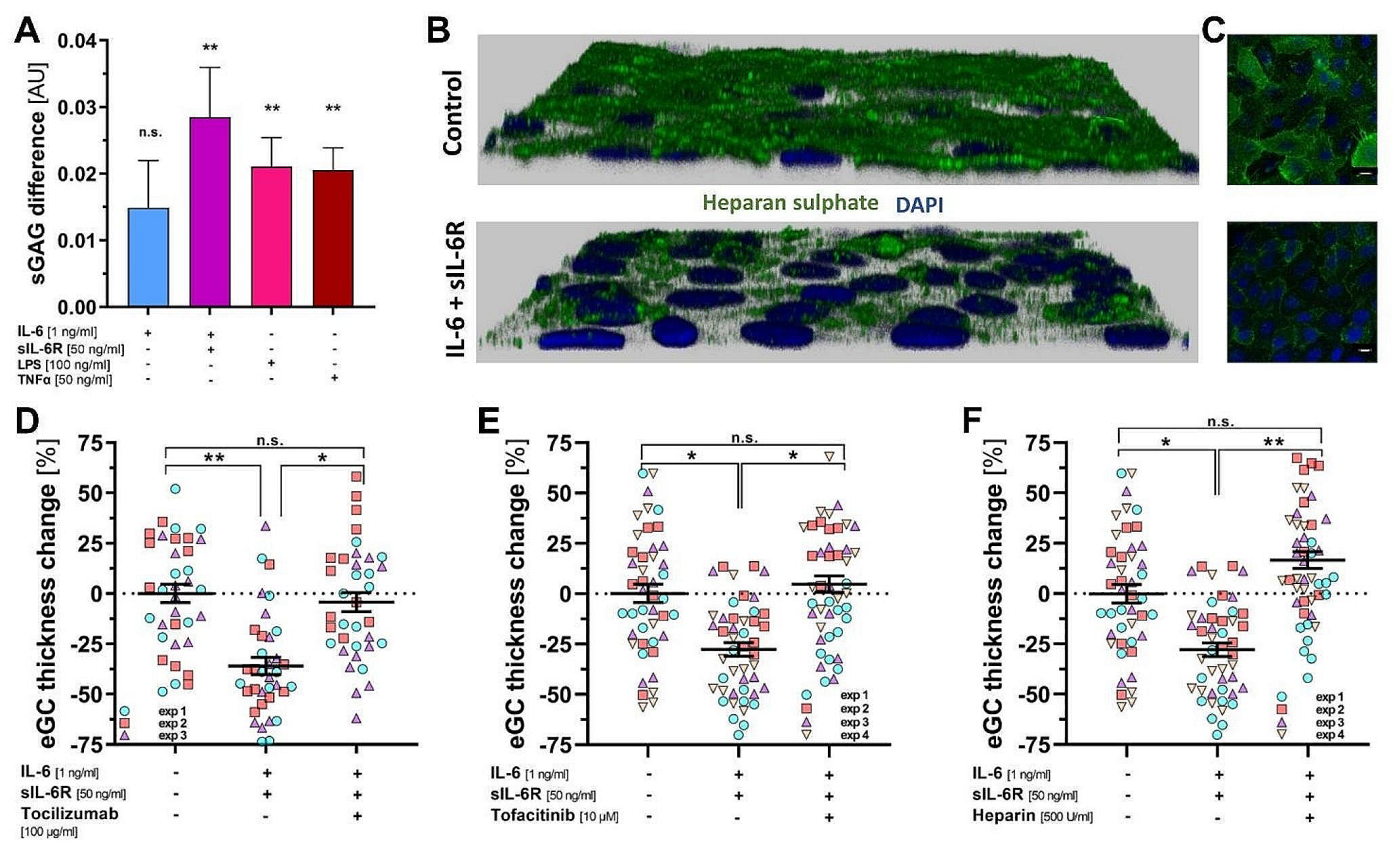
IL-6 trans-signalling causes eGC damage in vitro. (**A**) The amount of sulphated glycosaminoglycans (sGAGs) in the supernatant of endothelial cells was measured using the 1,9-dimethylmethylene blue (DMMB) assay. Cells were incubated for 60 min with the indicated concentrations of IL-6 ± sIL-6R, TNFα or LPS supplemented with CD14 (10 ng/ml) and LBP (100 ng/ml). Data are expressed as mean ± SEM of duplicates, *n* = 3–9. (**B** + **C**) Immunofluorescence imaging showing the distribution and coverage of heparan sulphate (green) staining on the cell layer in 3D reconstruction (**B**) derived from 2D images (**C**). Nuclei were stained with DAPI (blue). Scale bar 10 μm. Incubation was performed under the same conditions as described in (**A**) with IL-6 (1 ng/ml) + sIL-6R (50 ng/ml). Incubation in Hepes buffer supplemented with 1% fetal calf serum was used as a control. (**D**-**F**) Nanoindentation experiments with atomic force microscopy (AFM) showing changes of eGC height on living endothelial cells after 60 min incubation with sIL-6R + IL-6 ± tocilizumab, tofacitinib or heparin. Incubation in Hepes buffer supplemented with 1% fetal calf serum was used as control. All conditions were pre-incubated for 24 h with vehicle or in case of tofacitinib treatment with tofacitinib. Data are presented as mean ± SEM, each point represents the average of ≥ 4 indentations per cell with a minimum of 8 cells per experiment, *n* = 3-4. Significance was tested by nested ANOVA followed by Tukey’s or Dunnett’s post-hoc test. *=*p* < 0.05, **=*p* < 0.01

Incubation of ECs with low to supra-physiological concentrations (0.1–10 ng/ml) of IL-6 alone resulted in a weak and dose-independent increase in sGAG content in the endothelial cell supernatant (data not shown), suggesting that damage to the eGC by classical signalling mechanisms is negligible. However, the addition of a physiologic concentration of sIL-6R (50 ng/ml) to simulate trans-signalling showed a doubling of the amount of sGAG in the supernatant in response to an intermediate concentration of IL-6, consistent with a more potent effect of trans-signalling (Fig. 2A). Accordingly, 2D images and 3D reconstructions of immunofluorescence-stained heparan sulphate, the major GAG of the eGC, showed that the intensity and coverage of the EC surface is reduced after incubation with IL-6/sIL-6R (Fig. 2B, C). As systemic trans-signalling dominates in acute inflammatory responses such as sepsis and COVID-19 [33], all further in vitro experiments were performed with the combination of IL-6 (1 ng/ml) and sIL-6R (50 ng/ml).

We then used the much more accurate nano-indentation AFM method to investigate clinically available inhibitors of IL-6 signalling in vitro. Co-incubation with the humanized monoclonal antibody tocilizumab (100 µg/ml), an antibody against the IL-6R that prevents IL-6 from binding to the IL-6R, completely prevented IL-6-induced glycocalyx damage on ECs (Fig. 2D). Similar results were obtained by blocking Janus kinases (JAKs), which act downstream of gp130, with tofacitinib (10 µM, pre-incubation for 24 h) (Fig. 2E).

We have previously shown that in sepsis and COVID-19, the final common pathway of eGC damage appears to be the activation and release of the heparan sulphate-degrading enzyme heparanase [11, 12]. The addition of heparin - a potent heparanase inhibitor - also protected eGC from IL-6-induced damage, suggesting that IL-6 signalling ultimately acts to regulate heparanase (Fig. 2F).

### Tocilizumab protects from serum-induced eGC damage in bacterial sepsis and COVID-19

**Fig. 3:**
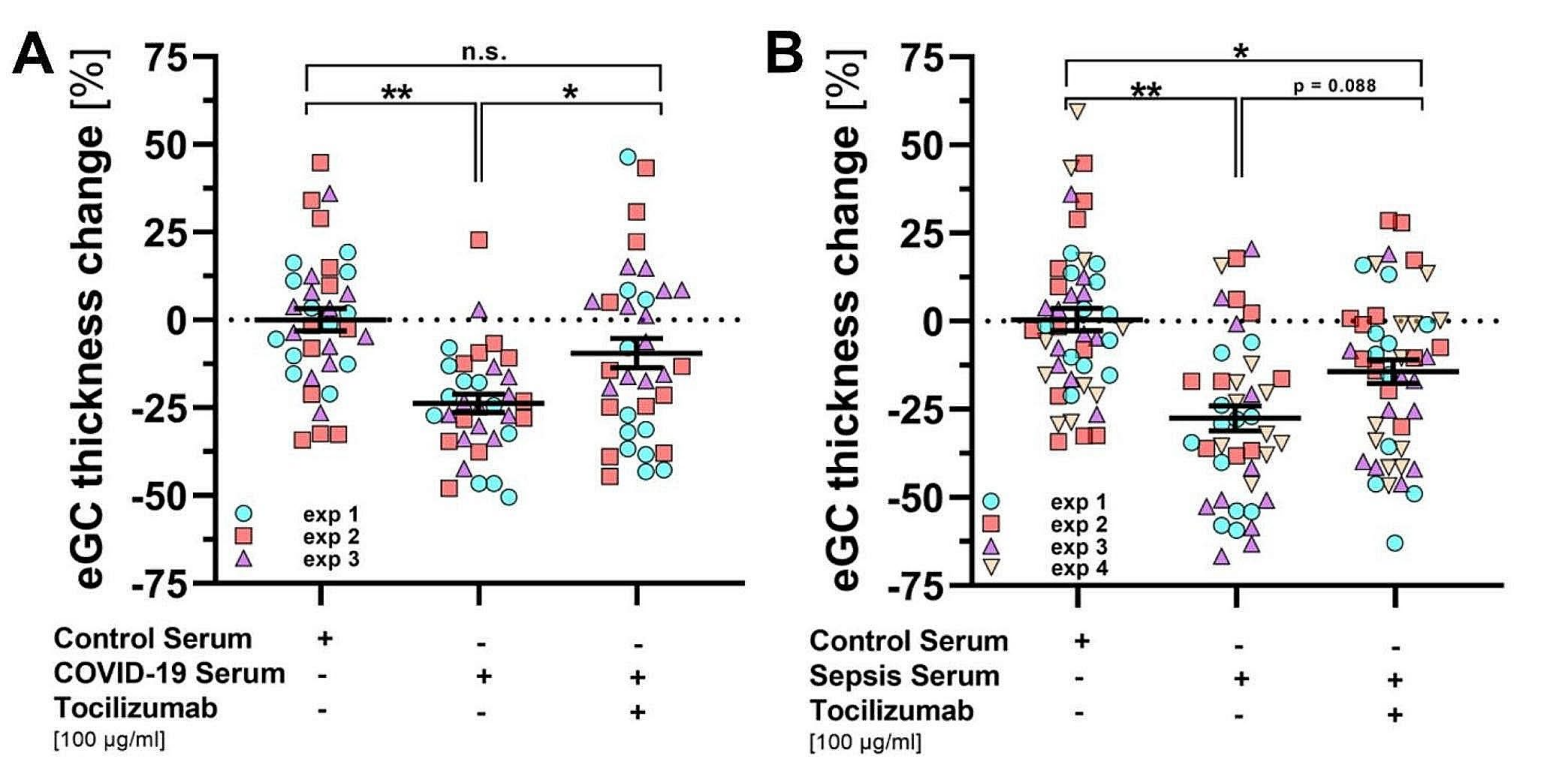
Tocilizumab protects from serum-induced eGC damage in bacterial sepsis and COVID-19. Nanoindentation experiments with atomic force microscopy (AFM) showing changes of eGC height on living endothelial cells after 60 min incubation with pooled serum samples (5%) from (**A**) COVID-19 and (**B**) sepsis patients with or without concomitant addition of tocilizumab. Pooled sera from healthy individuals were used as control. Data are presented as mean ± SEM, each point represents the average of ≥ 4 indentations per cell with a minimum of 8 cells per experiment, *n* = 3–4. Significance was tested by nested ANOVA followed by Tukey’s post-hoc test, *=*p* < 0.05, **=*p* < 0.01

To simulate more realistic inflammatory conditions in the context of sepsis and COVID-19, three randomly selected serum samples from the third tertile (highest IL-6 concentrations) of each COVID-19 and sepsis were pooled and used for the following in vitro experiments. Mean [± SEM] IL-6 concentrations in the pooled sepsis subgroup were higher than in the COVID-19 subgroup (2859 [± 1275] vs. 636 [± 476] ng/ml). Incubation of ECs with pooled serum (5% for 60 min) resulted in a significant decrease in eGC in both sepsis and COVID-19. Co-incubation with tocilizumab (100 µg/ml) protected the eGC from COVID-19 serum-induced damage (Fig. 3A). A similar trend was observed with co-incubation of tocilizumab with sepsis serum, although it was not statistically significant (Fig. 3B).

### Proteome-derived eGC_signature_ correlates with IL-6 and outcome in external validation set

**Fig. 4:**
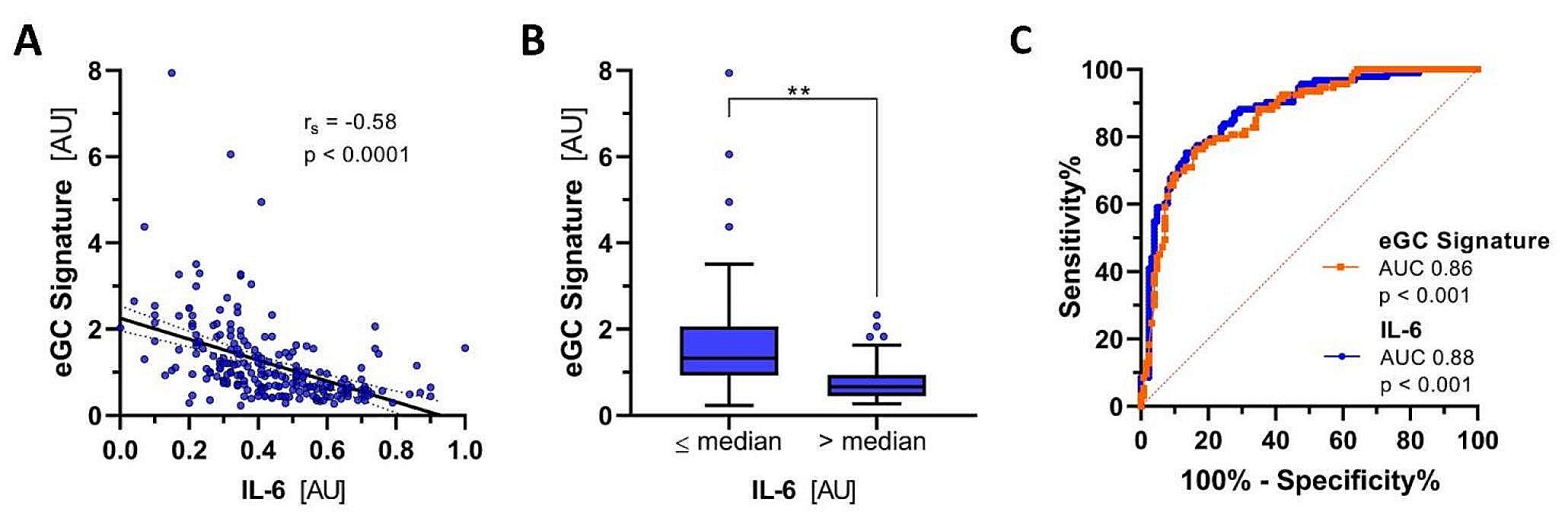
Proteome-derived eGC_signature_ correlates with IL-6 and outcome in external validation set. External validation of a previously identified proteomic signature that correlates well with eGC thickness (hereafter referred to as eGC_signature_) in an independent COVID-19 cohort from Massachusetts General Hospital (*n* = 219). (**A**) Dot plot analysis showing the correlation between the eGC_signature_ and IL-6. (**B**) eGC_signature_ classified based on median IL-6 levels. (**C**) Receiver operating characteristic curves showing the predictive ability of eGC_signature_ and IL-6 to predict the composite endpoint of 28-day mortality and/or intubation. AU arbitrary units. **p < 0.01

In the last step, we wanted to validate the mechanistic link between PBR and IL-6. As our study was not designed for outcome analysis, we used the external MGH COVID-19 cohort, which annotates proteome and outcome data. As sublingual microscopy was not performed in the MGH cohort, we used a previously validated proteomic signature that correlates well with eGC thickness as a surrogate (hereafter referred to as eGC_signature_) [15]. Patients’ eGC_signature_ values correlated inversely well with IL-6 levels (r_s_ = -0.58, *p* < 0.0001) (Fig. 4A). When dichotomized by the median, patients with lower IL-6 levels had a higher eGC_signature_, values indicating a healthier eGC (*p* < 0.0001) (Fig. 4B). For the composite endpoint of 28-day mortality and/or intubation, eGC_signature_ performed similarly to IL-6 (AUC [95% CI] 0.86 [0.81–0.91], *p* < 0.001] vs. IL-6 0.88 [0.83–0.92], *p* < 0.001), further supporting the proposed mechanistic role of IL-6 in eGC damage (Fig. 4C).

## Discussion

This study demonstrates that IL-6 and its downstream signalling have a causal role in eGC damage. In vitro pharmacological IL-6 blockade protected against eGC damage induced by sera from bacterial sepsis and COVID-19 patients. Additionally, IL-6 levels correlated with features of eGC impairment and predicted outcomes in an external COVID-19 cohort. These data clearly suggest that IL-6 may be a significant driver of eGC damage during systemic inflammation.

A first hint suggesting a possible link between IL-6 and the eGC came from Ikonomidis et al. who observed a decrease of the PBR (i.e. improvement of the eGC) upon tocilizumab administration in patients with rheumatoid arthritis [34]. In the case of COVID-19, evidence of eGC damage has been found in numerous studies [35–37]. However, the pathophysiological pathways that trigger this damage are not yet fully understood.

By establishing a causal role for IL-6 in eGC damage, we add an important piece of the puzzle to the existing literature. Therapeutic strategies targeting IL-6 have been successful in the treatment of severe COVID-19 disease, making this finding even more exciting, and regulatory authorities have recently approved its therapeutic use [38–42]. A recent meta-analysis [43] showed that IL-6 blockade with tocilizumab works best for moderate to severe COVID-19. Contrary to expectations, secondary infections were not increased with the administration of IL-6 receptor antagonists in COVID-19 patients. However, IL-6R blockade increases the risk of bacterial, viral, and opportunistic infections in rheumatoid arthritis [44] and should therefore not be used in bacterial sepsis.

In our cell culture model, blocking not only binding of IL-6 to IL-6R, but also its downstream JAK/STAT pathway with tofacitinib was sufficient to counteract eGC damage. Clinical studies on hospitalized COVID-19 patients have also demonstrated a survival benefit for this class of substances [45–47]. However, as none of the clinical trials on anti-IL-6 therapy in COVID-19 used sublingual microscopy to estimate the eGC properties, we are currently unable to estimate the effect of anti-IL-6 treatment on eGC dimensions in vivo. However, due to the widespread systemic vascular involvement in COVID-19, it appears plausible that anti-IL-6 therapy would have reduced damage to the eGC. This hypothesis is further supported by the strong correlation between IL-6 and eGC signature in the validation cohort.

Although it is currently unclear whether IL-6 inhibition has similar benefits in sepsis, a recent Mendelian randomization analysis suggests that IL-6 receptor blockade is associated with lower mortality in 11,643 sepsis patients of the UK Biobank cohort [48]. Barkhausen et al. reported that in a murine polymicrobial sepsis model, pretreatment with a selective inhibitor of IL-6 trans-signalling increased survival from 45 to 100% in a dose-dependent manner [49]. Similarly, specific inhibition of IL-6 trans-signalling completely prevented death in mice with endotoxic shock [50]. Kang et al. also detected improved survival in a murine endotoxic shock model by counteracting vascular injury upon treatment with anti-IL-6R antibody. They could further demonstrate in a murine burn injury model, that short-term IL-6R inhibition preserved eGC integrity in capillaries visualized by electron microscopy [51]. These studies support the results of our in vitro experiments and emphasize the central importance of IL-6 in bacterial sepsis, which is also reflected in its prominent role as an early diagnostic and prognostic biomarker [52–54].

However, it is important to note that the preventative effect of tocilizumab on serum-induced eGC damage was somewhat weaker in bacterial sepsis compared to COVID-19. It is unlikely that this is only due to a pure dose effect of IL-6 (which is about four times higher in sepsis), as tocilizumab was administered in a saturating dose. It is more likely that eGC in sepsis is affected by additional and/or more complex mechanisms, which may explain the lack of clinical efficacy of several targets that (at least in theory) should also diminish heparanase release [55]. Further research is required to determine the role of other harmful mediators in the complex cytokine milieu during systemic inflammation. The aim should be to identify the smallest possible set of key mediators or effectors whose blockade can prevent eGC damage in a non-redundant manner. In the case of COVID-19, the singular blockade of IL-6 already appears to be close to achieving this goal.

As this study is primarily hypothesis-generating, it is important to note some limitations. Firstly, the sample size of this cross-sectional study was rather small and was not suitable for the analysis of clinical outcomes. However, the comprehensive dataset combines serum proteomics with intravital microscopy. Secondly, it was not our intention to make a direct comparison between the two entities, but rather to analyze IL-6 in the context of systemic inflammation, using sepsis and COVID-19 as prototypical diseases. Although the entities were initially pooled, the supergroup analyses clearly show that IL-6 appears to play an important role in both. Thirdly, although routine microbiological sampling was performed in all patients, we cannot exclude the possibility of bacterial superinfections in the COVID-19 group, which may have partially influenced IL-6 levels. However, the prevalence of bacterial co-infections in COVID-19 is considered rather low and our COVID-19 cohort had a low median PCT value (0.6 ng/ml), which argues against overt co-infections. Fourthly, measuring the eGC both in vivo and in vitro presents a challenge due to the fragility of this delicate layer. Therefore, we complemented intravital microscopy and atomic force microscopy with additional detection methods, such as immunofluorescence and ELISA measurements. These methods broadly confirmed the microscopy data. However, it is crucial to acknowledge that determining PBR through intravital microscopy and measuring eGC thickness using AFM have limitations, which must be considered when interpreting the results. The AFM method may only detect the denser parts of the eGC. However, a strong correlation between the two methods has been observed multiple times using matched data [12, 19]. A detailed description of the advantages and disadvantages of these and other methods was published recently [4]. Finally, it is possible that randomly selecting and pooling of 3 sera from the upper percentile of each group for in vitro experiments may have introduced bias. However, this approach has been a valid compromise in our previous AFM studies [12, 19].

## Conclusion

Our data reveal a novel mechanistic pathway elucidating endothelial glycocalyx damage in inflammatory states such as bacterial sepsis and COVID-19. Further in vivo studies should validate our findings and demonstrate the protective efficacy of potential therapeutic interventions targeting IL-6 signalling on the endothelial glycocalyx. These trials will not only improve our pathomechanistic understanding but also pave the way for targeted interventions.

### Electronic supplementary material

Below is the link to the electronic supplementary material.

Supplementary Material 1

## Data Availability

The datasets used and/or analyzed during the current study are available from the corresponding author on reasonable request.

## Abbreviations

AU: Arbitrary unit
AUC: Area under the curve
COVID-19: Coronavirus disease 2019
CRP: C-reactive protein
DMMB: 1,9-Dimethylmethylene Blue
PCT: Procalcitonin
EC: Endothelial cell
eGC: Endothelial glycocalyx
HPSE: Heparanase
HS: Heparan sulphate
HUVEC: Human umbilical vein endothelial cell
ICU: Intensive care unit
IL-6: Interleukin-6
IL-6R: Interleukin-6-receptor
IMC: Intermediate care
IQR: Interquartile range
PBR: Perfused boundary region
RBC: Red blood cell
SEM: Standard error of the mean
sGAG: Sulphated glycosaminoglycan
SOFA: Sequential organ failure assessment
TLR: Toll-like receptor
TNF: Tumor necrosis factor
