# Supplementary material for: Interleukin-6 drives endothelial glycocalyx damage in COVID-19 and bacterial sepsis
Source: Angiogenesis. 2024 Apr 10;27(3):411–22. doi: 10.1007/s10456-024-09916-w (PMC11303473; doi:10.1007/s10456-024-09916-w)
Supplement: Supplementary file 1 — Supplementary Material 1 [file 10456_2024_9916_MOESM1_ESM.docx]

**Interleukin-6 drives endothelial glycocalyx damage in COVID-19 and bacterial sepsis**

**Running title:** IL-6 drives eGC damage

Carolin Christina Drost^1^, Alexandros Rovas^1^, Irina Osiaevi^1,2^, Klaus Schughart^3,4^, Alexander Lukasz^1^, Wolfgang A. Linke^5^, Hermann Pavenstädt^1^, Philipp Kümpers^1^


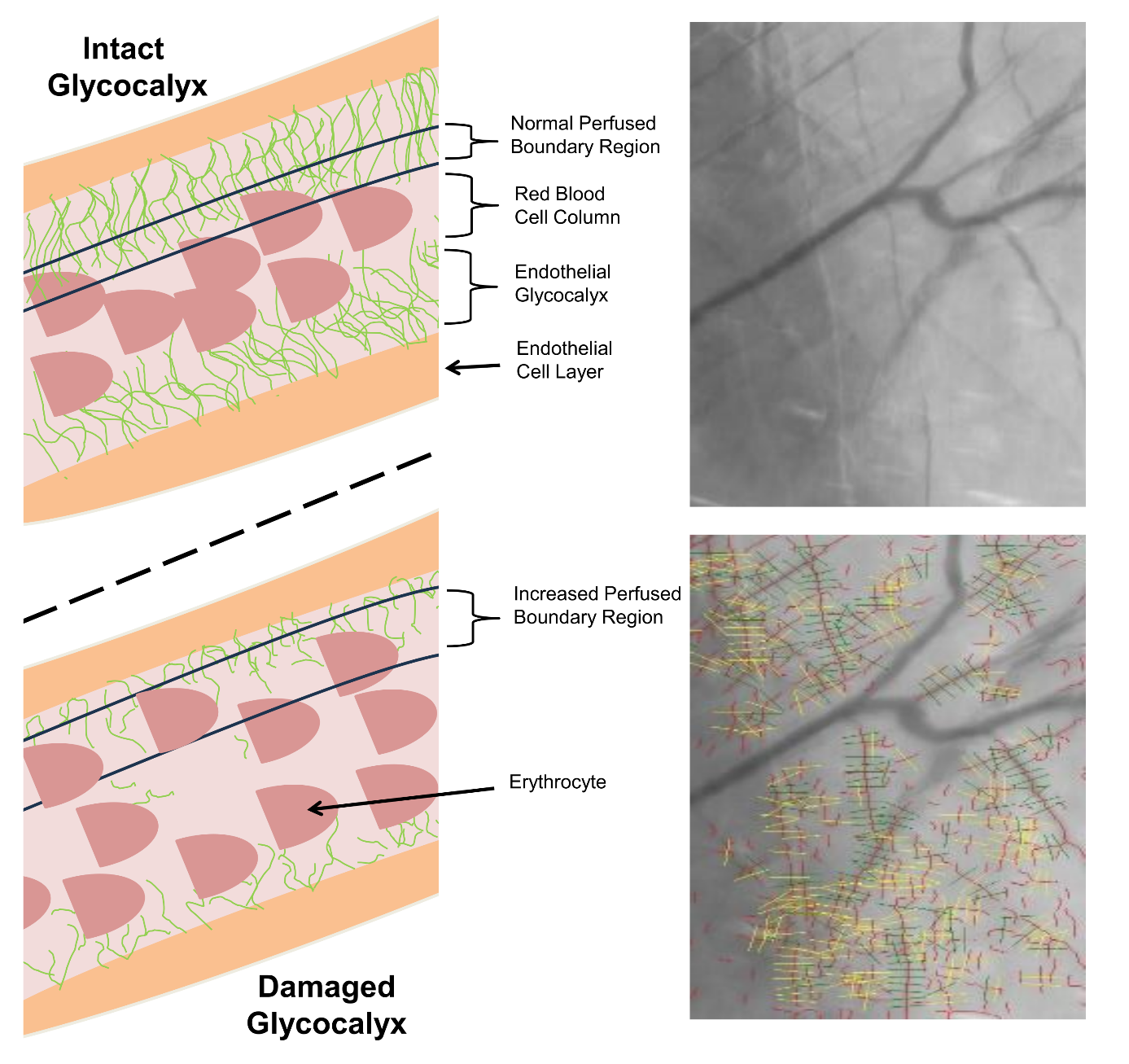


**Supplemental Figure 1: *Schematic of glycocalyx assessment by sublingual video microscopy.***

**Left:** Damaged eGC allows an increasing lateral drift of erythrocytes towards the endothelium. This results in an increased perfused boundary region (PBR, area between black lines). **Right:** Representative images from the dedicated GlycoCheck™ system. The software automatically excludes invalid segments (red, yellow). Adapted from Drost et al**.**, modified [1].


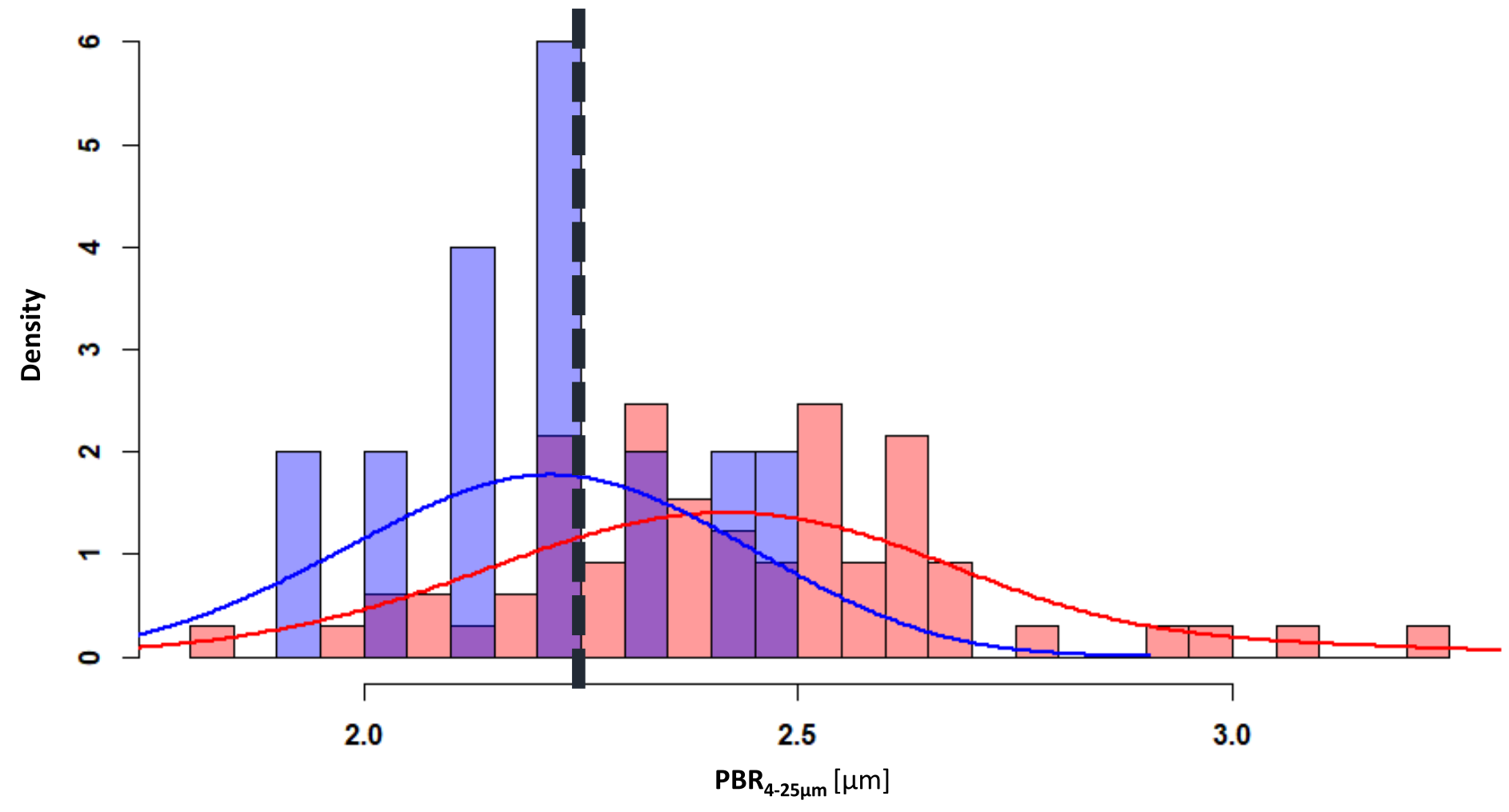


**Supplemental Figure 2: *PBR cut-off derivation.***

The histogram of the PBR distribution in the current cohort is shown separately for healthy controls (violet) and patients (red). As the PBR values of healthy controls are likely to be distributed around a 'normal' baseline PBR, we decided to use this collective as a basis to define a cut-off into low and high PBR for further analysis. Half of the healthy collective, including the maximum peak, was defined as providing low PBR values. Consequently, a PBR of 2.25 µm served as the cut-off used to divide the entire cohort into low and high PBR collectives, independent of disease status.


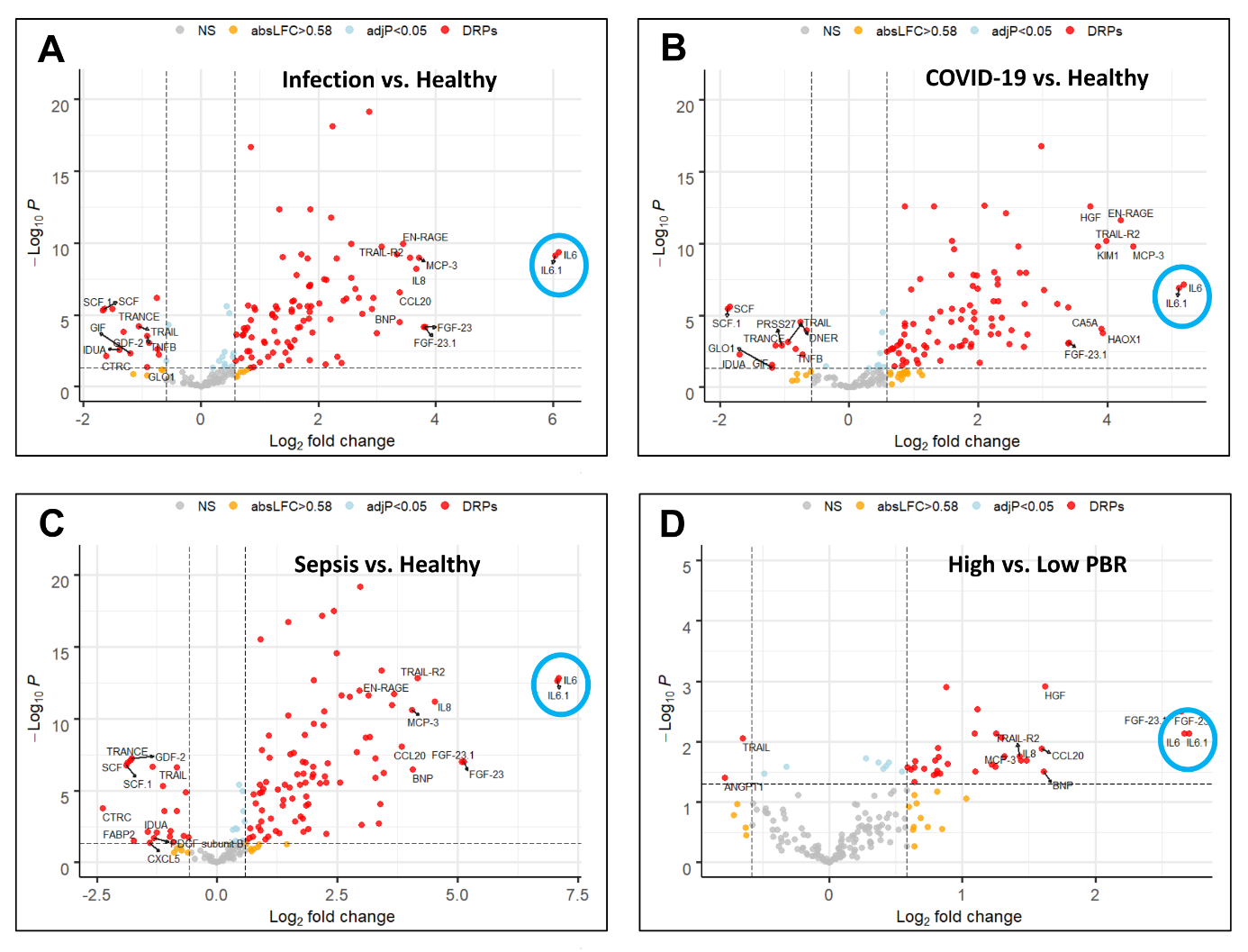


**Supplemental Figure 3: *Proteome analysis in different subgroups***

Volcano plots showing log_2_-fold changes and adjusted p-values of differentially expressed proteins (DEPs) from **(A)** the contrast of all infected (bacterial sepsis and COVID-19 combined, n=65) versus healthy controls (n=10), **(B)** the contrast of healthy controls (n=10) vs. COVID-19 ICU (n=15), **(C)** the contrast of healthy controls (n=10) vs. bacterial sepsis ICU (n=33), **(D)** the contrast of participants with high PBR (>2. 25, n=52) versus low PBR (≤ 2.25, n=23). The top 10 DEPs by log-fold change are labelled. As IL-6 is included in both proteomic panels, it appears twice. NS: not significant. absFC: absolute fold-change, adjP: adjusted p-value, DRPs: differentially expressed proteins. Note that Fig. S3D is identical to Fig. 1B.


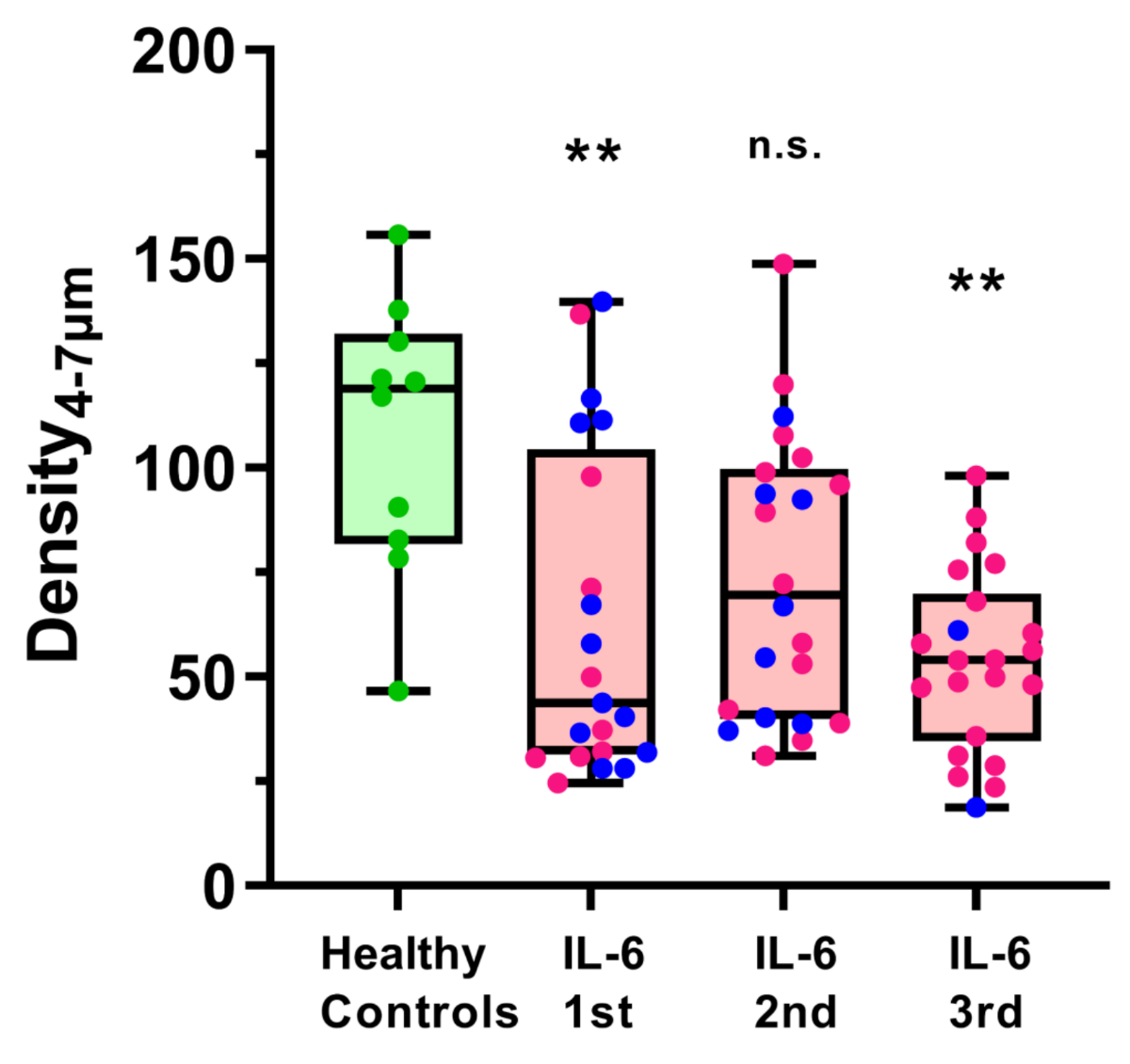


**Supplemental Figure 4: *Capillary density according to IL-6 tertiles.*** Box plots (median ± IQR) of capillary density (microvessels with a diameter of 4-7 µm in 10^-2^mm/mm^2^) values from sublingual video microscopy in healthy controls vs. patients (tertiles derived from IL-6, first tertile = lowest IL-6 levels). Individual values are shown as green dots = healthy controls; blue dots = COVID-19; pink dots = sepsis. Significance was tested with Kruskal-Wallis and Dunn's post-hoc test against healthy controls. *=p<0.05, **=p<0.01

**Supplemental Tables**

**Supp. Table 1**: *Baseline characteristics*

| **Variables** | **Healthy Controls** | **IMC** | **ICU** | **p value*** |
| --- | --- | --- | --- | --- |
| Number of participants (n) | 10 | 17 | 48 | - |
| COVID-19 (n; (%)) | - | 7 (41.2) | 15 (31.3) | 0.46 |
| Female sex (n; (%)) | 7 (70) | 8 (47.1) | 9 (18.8) | **0.023** |
| Age (years, median (IQR)) | 51 (27 – 69) | 64 (55 – 79.5) | 64.5 (56.3 – 76.5) | 0.69 |
| BMI (kg/m^2^, median (IQR)) | 23 (21.5 – 25.8) | 24.3 (21.2 – 27.5) | 25.8 (22.6 – 29.4) | 0.28 |
| Charlson Comorbidity Index  (points, median (IQR)) | - | 2 (0,5-5) | 2 (0-3) | 0.55 |
| SOFA score (points, median (IQR)) | - | 2 (0.5 – 3) | 10 (6 – 13) | **<0.0001** |
| Mechanical ventilation (n; (%)) | - | 0 (0.0) | 32 (66.7) | **<0.0001** |
| Inhospital mortality (n; (%)) | - | 2 (11.8) | 17 (35.4) | 0.065 |
| MAP (mmHg, median (IQR)) | 92.3 (89.2 – 99.4) | 85.3 (71.7 – 104) | 73.5 (67.4 – 84.6) | **0.008** |
| **Sublingual video-microscopy** (median (IQR)) |  |  |  |  |
| PBR_4-25µm_ (µm) | 2.23 (2.1 – 2.34) | 2.3 (2.18 – 2.48) | 2.47 (2.32 – 2.62) | **0.023** |
| RBCV_4-7µm_ (µm/sec) | 100 (88 – 118) | 108 (104 – 120) | 82 (76 – 97) | **<0.0001** |
| Density_4-7µm_ (10^-2^mm/mm^2^) | 118.9 (81.7 – 132.1) | 53 (37.4 – 91.5) | 57.1 (36.7 – 91.6) | 0.92 |
| **Laboratory data** (median (IQR)) | | | |  |
| CRP (mg/dl) | 0.5 | 10 (1.6 – 18.5) | 21.7 (13.7 – 33) | **0.0002** |
| PCT (ng/ml) | 0.05 | 0.21 (0.11 – 0.67) | 5.58 (1.14 – 39.69) | **<0.0001** |
| Creatinine (mg/dl) | 0.85 (0.68 – 0.95) | 1.1 (0.68 – 1.9) | 1.8 (0.9 – 3.1) | 0.07 |
| IL-6 (ng/ml) | 2 | 41 (25 – 233) | 203 (84 – 865) | **0.008** |

*p-value was calculated between IMC and ICU cohort. Analysis was performed with Mann-Whitney test or Chi-square test as appropriate. BMI = Body mass index, CRP = C-reactive protein, IQR = interquartile range, MAP = Mean arterial pressure, PCT = Procalcitonin, SOFA score = Sequential Organ Failure Assessment score, PBR = Perfused boundary region, RBCV = Red blood cell velocity, IL-6 = Interleukin-6

**Supp. Table 2**: *Participants’ characteristics according to eGC status*

| **Variables** | **low PBR (≤2.25)** | **high PBR (>2.25)** | **p value** |
| --- | --- | --- | --- |
| Number of participants (n) | 23 | 52 | - |
| Female sex (n; (%)) | 8 (35.8) | 16 (30.8) | 0.79 |
| Age (years, median (IQR)) | 62 (50 – 69) | 66 (57 – 77) | 0.04 |
| BMI (kg/m^2^, median (IQR)) | 23.4 (21.3 – 26.5) | 25.9 (22.6 - 28.9) | 0.14 |
| ICU (n; (%)) | 8 (34.8) | 40 (76.9) | 0.0005 |
| Mechanical ventilation (n; (%)) | 5 (21.7) | 27 (51.9) | 0.02 |
| **Sublingual video-microscopy** |  |  |  |
| PBR_4-25µm_ (µm) | 2.16 (2.05 – 2.23) | 2.5 (2.38 – 2.62) | <0.0001 |
| RBCV_4-7µm_ (µm/sec) | 101 (83 – 114) | 91 (78 – 107) | 0.17 |
| Density_4-7µm_ (10^-2^mm/mm^2^) | 54.0 (37.1 – 111.4) | 59.3 (39.3 – 93.4) | 0.90 |
| **Laboratory data** (median (IQR)) | | | |
| CRP (mg/dl) | 3.2 (0.5 – 15.3) | 21.4 (11.5 – 31.1) | <0.0001 |
| PCT (ng/ml) | 0.12 (0.05 – 0.76) | 3.22 (0.64 – 22.50) | 0.0001 |
| Creatinine (mg/dl) | 0.9 (0.7 – 1.3) | 1.6 (0.83 – 3.1) | 0.04 |
| IL-6 (ng/ml) | 30 (2 – 90) | 131 (69 – 826) | 0.0003 |

Analysis was performed with Mann-Whitney test or Chi-square test as appropriate.

BMI = Body mass index, CRP = C-reactive protein, IQR = interquartile range, MAP = Mean arterial pressure, PCT = Procalcitonin, SOFA score = Sequential Organ Failure Assessment score, PBR = Perfused boundary region, RBCV = Red blood cell velocity, IL-6 = Interleukin-6

**Literature**

1. Drost CC, Rovas A, Kumpers P: **Protection and rebuilding of the endothelial glycocalyx in sepsis - Science or fiction?** *Matrix Biol Plus* 2021, **12**:100091.
